# Autoimmune Encephalitis With Autoimmune Diabetes: A Case of Horror Autotoxicus

**DOI:** 10.7759/cureus.34268

**Published:** 2023-01-27

**Authors:** Hussam Alkaissi, Jung-Hyun Lee, Samy I McFarlane

**Affiliations:** 1 Internal Medicine, Kings County Hospital Center, Brooklyn, USA; 2 Internal Medicine, Veterans Affairs Medical Center, Brooklyn, USA; 3 Internal Medicine, State University of New York Downstate Health Sciences University, Brooklyn, USA; 4 Neurology, Kings County Hospital Center, Brooklyn, USA

**Keywords:** glutamic acid decarboxylase (gad) antibodies, type 1 diabetes mellitus (t1d), autoimmunity, autoimmune encephalitis, seronegative autoimmune encephalitis

## Abstract

Diagnosing autoimmune encephalitis relies on clinical, radiological, and serological studies. Several autoantibodies have been implicated and recognized, with dozens of potential targets identified in the past 20 years. Despite that progress, some patients with encephalitis present a diagnostic dilemma with a seronegative status. The presence of other autoimmune diseases in a patient with encephalitis should provide a clue to the autoimmune nature of a developing neurological syndrome (cognitive, psychiatric, behavioral, and catatonia). In this report, we describe the case of a young man with type 1 diabetes mellitus who was diagnosed with seronegative autoimmune encephalitis after presenting with catatonia. We describe the lengthy clinical course, the various therapeutic trials, and his clinical outcome and response to B-cell depleting agent.

This study also discusses the potential pathophysiologic pathways, providing a rationale for the diagnostic workup and therapeutic options for autoimmune encephalopathy in this case presentation.

## Introduction

Autoimmune encephalitis (AE) manifests with a wide range of neuropsychiatric symptoms, and depending on the antibody associated, clinical and immunologic features may vary [1]. Diagnosis of AE currently relies on clinical suspicion, associated autoantibodies in serum or cerebrospinal fluid (CSF) analysis, pertinent findings on imaging studies, electroencephalogram (EEG), and response to immunotherapy [2]. Establishing a prompt diagnosis and initiating early treatment is essential in preventing the potential complications of this serious illness. In the absence of characteristic antibodies, however, diagnosis may be challenging, and treatment may be delayed. Here we describe a case of a 33-year-old male with type 1 diabetes mellitus (T1DM) who initially presented with catatonia and was subsequently diagnosed with seronegative AE.

## Case presentation

A 33-year-old male with a recent diagnosis of type 1 diabetes mellitus (T1DM) was hospitalized with a change in mental status. The patient was found by his roommate to be unresponsive in his bed, awake, eyes opened but unable to move or communicate. Six months before admission, he was admitted for diabetic ketoacidosis, and diagnosed with T1DM, with low titer positive anti-glutamic acid decarboxylase (GAD) antibodies, after which he was discharged on subcutaneous insulin regimen of short and long-acting insulin analogs (aspart and glargine insulin). He had a past medical history of alcohol use, with daily or every other day drinking of one to three beers, with no history of illicit drug use, and no psychiatric history. There was no recent travel history or exposure to animals or pets. His family history and occupational history were non-contributory.

**Figure 1:**
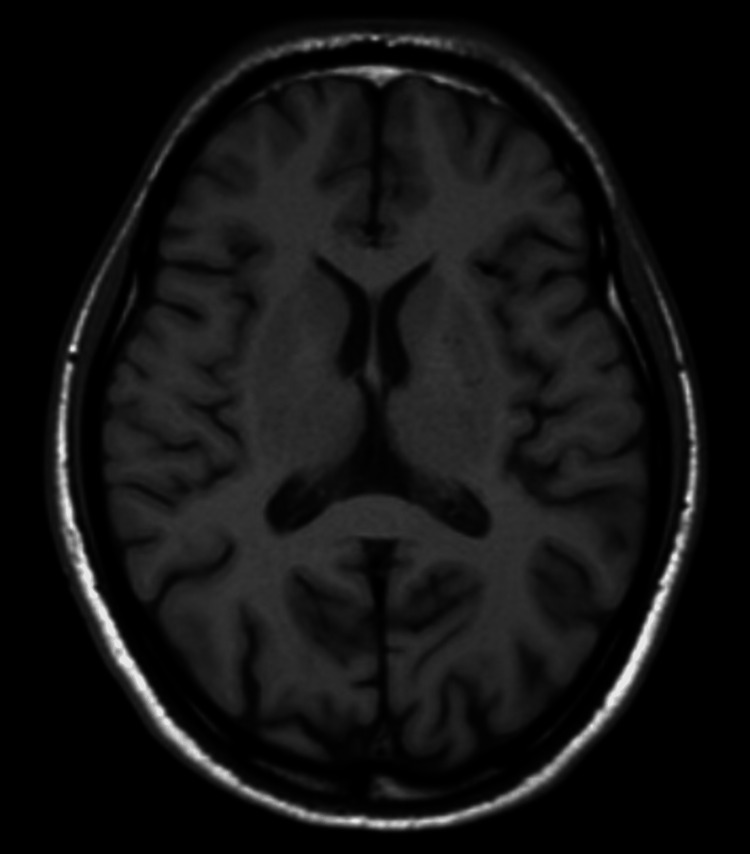
Normal brain MRI (T1-weighted imaging) with no mass effect. Relative volume loss is seen in other sections.

On physical examination, the patient was hemodynamically stable, afebrile, alert, and in a catatonic state, with eyes opened but unable to express himself or follow commands. He had minimal spontaneous movement from side to side, with slow movements of his extremities, but was unable to maintain a fixed posture when tested. The rest of the physical examination was unremarkable. The initial laboratory profile was unremarkable, with normal glucose levels (Table 1). Imaging showed cerebral atrophy relative to age but no mass lesion or abnormal intensity on T2-weighted imaging (Figure 1).

**Table 1:** Routine blood work, comprehensive metabolic panel, and complete blood count over a period of two years. NA: not available.

Variable	Day 1 of admission	1 month after admission	3 months after admission	6 months after admission	1 year after hospitalization	2 years after hospitalization	Reference range
Sodium (mmol/L)	141	136	134	136	142	138	135-145
Potassium (mmol/L)	3.8	4.2	4.4	4.7	4.4	4.4	3.5-5.1
Chloride (mmol/L)	99	98	101	97	104	100	98-107
Carbon dioxide (mmol/L)	21	24	26	30	29	30	21-31
Urea nitrogen (mg/dL)	14	6	9	10	10	9	7-25
Creatinine (mg/dL)	0.71	0.63	0.7	0.6	0.7	0.7	0.6-1.2
Calcium (mg/dL)	9.8	9.7	8.7	9.6	9.8	9.4	8.2-10
Albumin (g/dL)	5.1	4.5	4.5	4.3	4.6	4.7	3.5-5.7
Total protein (g/dL)	7.7	6.8	5.6	6.7	6.5	7.2	6-8.3
Aspartate aminotransferase (U/L)	42	55	34	20	24	22	10-35
Alanine aminotransferase (U/L)	30	151	43	64	44	20	0-31
Total bilirubin (mg/dL)	1.1	0.5	1.3	0.6	0.5	0.4	0-1.2
Alkaline phosphatase (U/L)	66	54	34	72	65	54	25-125
White-cell count (per µL)	4,900	2,100	4,800	2,350	3,970	2,100	3,500-10,800
Hemoglobin (g/dL)	13.7	12.6	12.7	11.3	12.8	13.5	12-16
Platelets count (per µL)	295,000	356,000	258,000	408,000	299,000	247,000	130,000-400,000
Ammonia (µmol/L)	31	NA	NA	NA	NA	NA	15-45

Lumbar puncture (LP) was done due to concern for meningoencephalitis, with initial cerebrospinal fluid (CSF) studies significant for elevated protein, with otherwise normal cell count, with no evidence of pleocytosis (Table 2). He had normal opening pressure, cytology, and polymerase chain reaction (PCR) for common pathogens were negative, as well as Gram stain, acid-fast stain, and cultures. No evidence of an oligoclonal band. Another sample was sent to test for autoantibodies.

**Table 2:** Lumbar puncture studies showed elevated protein on two occasions. *Polymerase chain reaction investigating *Escherichia coli*, *Haemophilus influenzae*, *Listeria monocytogenes*, *Neisseria meningitidis*, *Streptococcus agalactiae*, *Streptococcus pneumoniae*, HSV-1, HSV-2, varicella-zoster virus (VZV), Epstein-Barr virus (EBV), Cytomegalovirus (CMV), human herpesvirus 6 (HHV-6), Enterovirus, Parechovirus, Cryptococcus. **Testing for the following autoantibodies: NMDA-R antibodies, LGI1-IgG, CASPR2-IgG, GAD65, GABA-B-R antibodies, AMPA-R antibodies, anti-neuronal nuclear antibodies (type 1, type 2, and type 3), anti-Purkinje cytoplasmic antibodies (type 1, type 2, and type 3), amphiphysin antibodies, CRMP-5-IgG, DPPX antibodies, GFAP antibodies, mGluR1 antibodies, anti-glial nuclear antibodies. ***Lyme serum and CSF serology, syphilis venereal disease research laboratory test (VDRL) and serology, human T-lymphotropic virus 1/2 (HTLV1/2) serology, HIV serology, tuberculosis interferon-gamma release assay (IGRA), AFS and culture, toxoplasmosis serology. ACE: angiotensin-converting enzyme; PCR: polymerase chain reaction; ANA: antinuclear antibodies; GAD: glutamic acid decarboxylase; AFS: acid-fast staining

Variable	On admission	Five months after admission	Reference range
Opening pressure (cmH_2_O)	13	15	10-20
Appearance	Clear	Clear	Clear
Protein (g/dL)	131	115	18-45
glucose (mg/dL)	109	80	45-65
Gram stain	No bacteria seen	No bacteria seen	Absent staining
Glucose serum:glucose CSF ratio	0.7	0.65	0.6
White-cell count (per µl)	0	0	<3
Flow cytometry	Acellular sample	Acellular sample	-
PCR^*^	Negative	Negative	Negative
Autoantibodies^**^	Negative	ANA, anti-GAD low titers	Negative
ACE (U/L)	26	NA	14-82
Infectious disease serologies^***^	Lyme band 41	Lyme band 41	-
IgG index	0.4	NA	<0.7

CSF was also tested for Lyme disease, and western blot was positive for anti-p41 IgG (band 41) that normally targets the flagellin protein of Borrelia, with equivocal IgM against the same band and equivocal anti-p93 IgG. Otherwise all other antibodies were negative (anti-p18, p28, p30, p31, p39, p45, p58, p66, and p93, both IgG and IgM) (Table 2). The serum was negative for Borrelia antibodies. The test for latent tuberculosis with interferon-gamma release assay was negative, test for syphilis with serum and CSF venereal disease research laboratory (VDRL) and serology (antibodies) was also negative. Electroencephalogram (EEG) showed a slow posterior dominant rhythm of 6-7 Hz, diffuse delta and theta slowing with occasional right temporal focal delta slowing and moderate diffuse cerebral dysfunction. These findings suggest moderate diffuse cerebral dysfunction, non-specific in etiology, and focal right temporal dysfunction.

Given the presentation of catatonia, the patient was started on lorazepam challenge (intramuscular injection of 2 mg) with no measurable response. With equivocal findings of single band (anti-p41) antibodies on western blot for Lyme disease, the patient was treated with ceftriaxone 2 g daily for 21 days without a clinically appreciable response.

Of note, he was also noticed to have cyclical neutropenia throughout his lengthy hospitalization course ranging from 2,100 cells/µL to 4,000 cells/µL (Table 1). He was also noticed to have chronic diarrhea, and workup revealed low stool elastase pointing to chronic pancreatitis. Infectious workup, duodenal biopsy, and celiac serology were negative. The patient clinically responded to replacement of pancreatic enzyme (pancrelipase).

Due to concern for seronegative autoimmune encephalitis, the score for antibody prevalence in epilepsy and encephalitis (APE2 score) was calculated. APE2 score was 4, calculated as the following: 1 point for rapid onset neurological syndrome within a year, 1 point for neuropsychiatrist manifestations (catatonia), and 2 points for markers of inflammation in the CSF (protein >50 g/dL). As such, the patient was treated with methylprednisolone 100 mg daily for five days (pulse steroids therapy) and intravenous immunoglobulin (IVIg) 0.4 mg/kg/day for five days. Six weeks later, given the lack of response, electroconvulsive therapy (ECT) was tried for 18 sessions, followed by another 12 sessions, with minimal response, as the patient could move but was still very slow and unresponsive. Next, haloperidol was added and titrated up to 5 mg daily. Given the lack of response, amantadine was then added and titrated up to 200 mg three times daily. Further testing was done for thyroid function, serum autoantibodies, heavy metals, ceruloplasmin, and protein electrophoresis, all of which were within the normal reference range (Table 3).

**Table 3:** Other tests including thyroid function test, heavy metals, and protein electrophoresis. SPEP: serum protein electrophoresis; TPO: thyroid peroxidase; TSH: thyroid-stimulating hormone

Variable	Value	Reference range
TSH (µIU/mL)	1.58	0.35-4.8
Free T4 (ng/dL)	1	0.93-1.7
anti-TPO antibodies (IU/mL)	12.2	<34
anti-thyroglobulin (IU/mL)	<20	<40
Ceruloplasmin (mg/dL)	28.9	15-30
Copper (µg/dL)	123	72-166
Zinc (µg/dL)	59	56-134
Ferritin (ng/dL)	277	100-320
Mercury (µg/dL)	negative	<15
Lead (µg/dL)	<1	<10
SPEP Gamma band (g/dL)	2.6	(0.6-1.6)
Kappa chain (mg/dL)	0.9	0.3-1.9
Lambda chain (mg/dL)	0.45	0.57-2.6
Kappa/lambda ratio	2	0.2-1.6

**Figure 2:**
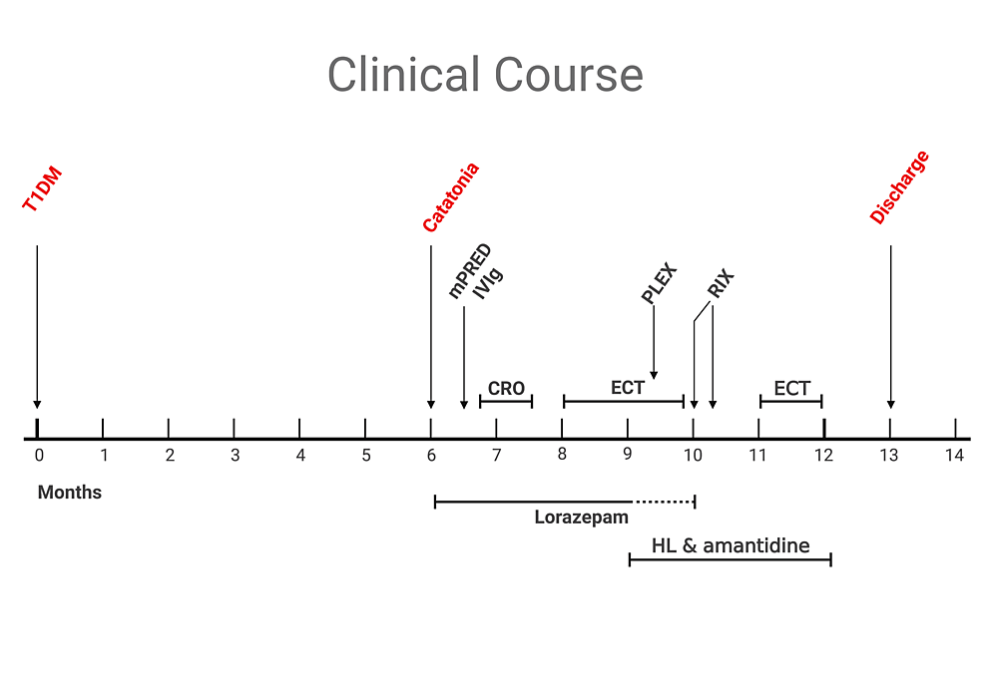
Clinical course of the patient. CRO: ceftriaxone; ECT: electroconvulsive therapy; HL: haloperidol; IVIG: intravenous immunoglobulin; mPRED: methylprednisolone; PLEX: plasma exchange; RIX: rituximab

LP was repeated with similar findings as previously described and was significant for only elevated opening pressure. CSF was tested for other antibodies and was positive for low titer of anti-GAD antibodies (0.07 nmol/L) and weak antinuclear antibodies (ANA), further raising the suspicion of an autoimmune process. Plasma exchange (PLEX) was introduced with no clinical response, after which a second-line treatment was considered. The patient was treated with two infusions of rituximab 1000 mg two weeks apart. After that, the patient started to respond to calling his name and gradually started answering questions, moving his extremities. In a few weeks, he was able to walk again. A month later, he was discharged to a rehabilitation facility (Figure 2). 

Six months later, the patient had two seizures during sleep. They were witnessed as shaking, unresponsiveness, and tongue biting, accompanied by stool incontinence, followed by a period of postictal state. Workup for seizures was unremarkable (normal EEG, MRI, and LP) and thus was empirically started on levetiracetam 1500 mg twice daily, with no recurrence of seizures. A year later, the patient was seen in the endocrinology clinic, able to walk, talk, express his needs, inject himself with insulin and perform his daily activities, with no recurrence of seizures or catatonic state. The clinical course is shown in Figure 2.

## Discussion

Our patient presented with neurological syndrome consisting mainly of catatonia six months after diagnosis with T1DM. In addition, cyclical neutropenia and diarrhea were noticed during the prolonged hospitalization course. Differential diagnoses were broad. Firstly, the combination of diabetes with neurological manifestations was initially concerning for a hypoglycemic encephalopathy. However, the prolonged irreversibility of the catatonia made hypoglycemia an unlikely diagnostic entity. In addition, findings on imaging studies were not suggestive of hypoglycemic encephalopathy, such as restriction on diffusion weight imaging (DWI) in white matter [3].

The combination of neurological syndrome with a recent diagnosis of diabetes prompted us to entertain the possibility of aceruloplasminemia, a monogenic disorder of impaired copper handling that leads to neurodegeneration with brain iron accumulation (NBIA) with iron accumulation in other organs, such as the pancreas leading to diabetes, often manifesting in middle age. The patient had a normal level of ceruloplasmin, copper, and iron studies. In addition, brain imaging ruled out any brain iron accumulation with normal susceptibility-weighted imaging (SWI) [4].

Adding diarrhea into the clinical picture further complicates the diagnostic approach. The differential diagnosis for encephalopathy and diarrhea may include the recently discovered anti-dipeptidyl-peptidase-like protein-6 (DPPX) encephalopathy. DPPX is a protein similar to DPP4 (CD26) and is expressed in the myenteric nervous plexus and the brain as part of voltage-gated potassium channel Kv4. Testing for anti-DPPX in CSF was negative (in addition to a battery of other autoantibodies) [5]. Whipple disease can manifest as diarrhea and CNS manifestations, so we had several intestinal biopsies, testing for periodic acid Schiff (PAS) stain, which was negative for Whipple disease. We also tested for celiac disease with anti-tissue transglutaminase (IgG and IgM), and both were negative with a normal duodenal biopsy. The cyclical neutropenia was unlikely to be part of the unifying diagnosis, as it was cyclical over two years and was also present even before presentation, with otherwise no evidence of immune deficiency; thus, it was likely a presentation of benign ethnic neutropenia [6]. Thinking of diagnosis based on clinical variables (i.e., CNS disease, diabetes, diarrhea) did not point to a final diagnosis. On the other hand, thinking of disease categories was more useful in this case.

Infectious causes were excluded by repeatedly negative CSF PCR, culture, and serologies, and empiric treatment for neuroborreliosis given a single band (anti-p41 IgG) with ceftriaxone did not yield in any clinical benefit and was further complicated by transient elevation of liver enzymes likely secondary to ceftriaxone treatment. The presence of anti-p41 is non-specific by itself; the antibody that naturally targets the flagellar protein of Borrelia species can cross-react with other viral, treponema infections, which were negative in our patient, and in some instances can be found even in healthy individuals, thus although sensitive, a single band on western blot for p41 antibodies is not a specific finding. Nevertheless, it may be a clue to an underlying humoral immunological process. Toxic and environmental exposures were unlikely, given that family members were unaffected and testing for heavy metals was negative. Genetic causes were also unlikely given a late presentation and negative testing for the few genetic disease biomarkers that can manifest at later age, namely with normal ceruloplasmin, ammonia level, and brain imaging.

Could it be autoimmune? A young, previously healthy individual, who was diagnosed six months before presentation with an autoimmune disease (type 1 diabetes), makes it possible that the neurological involvement was also autoimmune and that both conditions are related to each other, an example of Occam's razor, rather than Hickam's dictum [7].

By examining the evidence for autoimmunity here, we find elevated protein in the CSF, a non-specific antibody (anti-p41 on western blot), a polyclonal humoral immune process in the serum, with elevated gamma globulin of 2.6 g/dL, with slight kappa restriction (kappa:lambda ratio of 2). Based on clinical presentation and initial CSF studies, APE2 score was 4, which has a sensitivity of 97% but a lower specificity of 78-84%. Initial testing for autoantibodies in the CSF was negative for a long list of autoimmune (and paraneoplastic) antibodies. On a repeated test, we found a low titer of antinuclear antibodies in the CSF, further pointing toward seronegative autoimmune encephalitis. Imaging of the chest, abdomen, pelvis, and scrotum was done to screen for any occult malignancy that might be triggering paraneoplastic autoimmune encephalitis; all were normal.

Autoimmune encephalitis is often suspected in patients with neuropsychiatric manifestations, including altered levels of consciousness, seizures, memory loss, cognitive deficits, movement abnormalities, dysautonomia, behavioral change, psychosis, and catatonia [2,8]. Among several types of AE, autoimmune catatonia is most seen in anti-N-methyl-D-aspartate receptor (NMDAR) encephalitis. The pathophysiology of autoimmune catatonia in AE may involve adaptive immunity, including antibodies and T-cells targeting certain antigens such as NMDAR, and gamma-aminobutyric acid-A receptor (GABA-AR) [9]. Northoff suggested a model for catatonic symptoms describing "horizontal modulation" by glutamatergic dysfunction of orbitofrontal-prefrontal/parietal cortical relation explaining the behavioral and emotional symptoms and "vertical modulation" by GABAergic dysfunction in basal ganglia mediated by orbitofrontal cortical deficits explaining the motor symptoms of catatonia [10].

**Figure 3:**
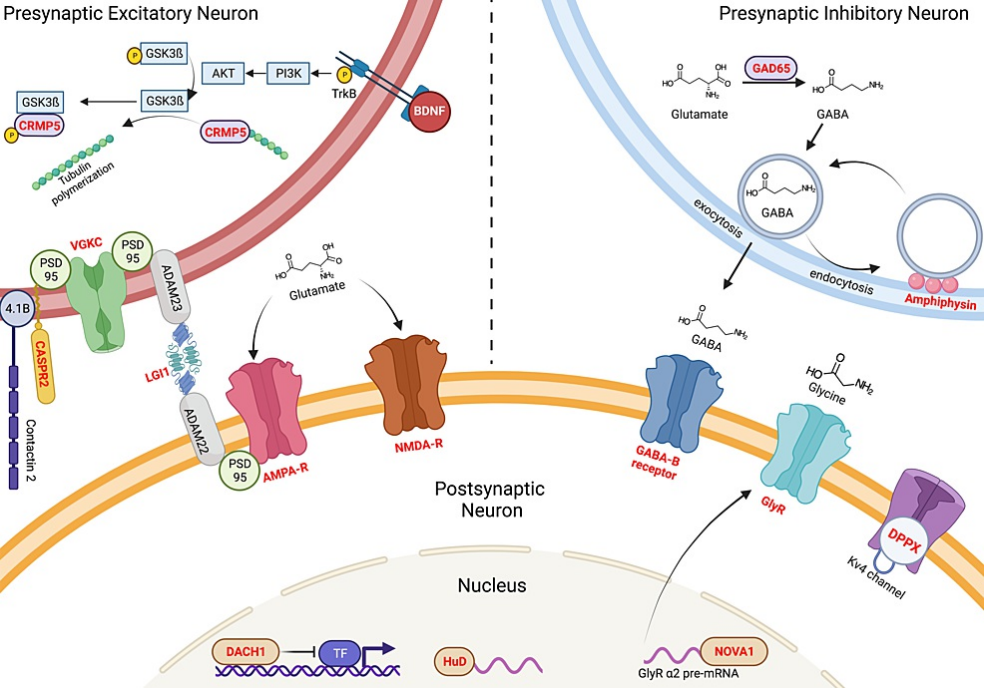
Several antigens are targeted by autoantibodies in AE. Some antigens are expressed in synapses such as channels, while others are intracellular (cytoplasmic or nuclear). Intracellular targets are shown in the postsynaptic neuron for illustration, but they can be found in any neuron, and in the case of DACH1, it is also expressed in breast cancer cells. AE: autoimmune encephalitis; DACH1: dachshund transcription factor 1; GSKß3: glycogen synthase kinase beta 3; PI3K: phosphoinositide 3-kinases

CSF in AE may show CSF pleocytosis, CSF-specific oligoclonal bands, or elevated CSF IgG index [2]. Autoantibodies against the intracellular antigen (Hu, Ma2, GAD65), synaptic receptor (N-methyl-D-aspartate receptors, AMPA, GABA-A/B, mGluR5, dopamine 2 receptor), ion channels (voltage-gated potassium channel-complexes), and other cell-surface proteins (leucine-rich glioma inactivated 1, contactin associated protein 2, dipeptidyl-peptidase-like protein-6, myelin oligodendrocyte glycoprotein, aquaporin 4, and GQ1b) are tested that may be directly or indirectly involved in the pathogenesis of AE [2]. Targets of these autoantibodies are shown in Figure 3 [11].

Autoantibodies target several antigens in AE. Some antigens are intracellular within the cytoplasm, such as glutamic acid decarboxylase 65 kDa (GAD), which removes the carboxyl group from glutamate, transforming it from an excitatory neurotransmitter to an inhibitory one, namely gamma-aminobutyric acid (GABA) which is stored in presynaptic vesicles. Released GABA binds to several GABA receptors that can be targeted in AE. Another protein that helps recycle vesicles is amphiphysin, which is also targeted in autoimmune encephalitis [12,13]. These autoantibodies (anti-GAD, anti-GABA-R, anti-amphiphysin) can result in hyperexcitability, rigidity, and stiff-person phenotype syndrome. Another cytoplasmic target of autoantibodies is collapsin response mediated protein 5 (CRMP5) which normally binds to tubulin protein, preventing its polymerization. However, upon the brain-derived neurotrophic factor (BDNF) binding to its receptor TrkB, subsequent second messengers (PI3K-Akt-GSK3ß) detach CRMP5 from tubulin, leading to tubulin polymerization and dendrites growth [14].

Another subset of intracellular targets is intranuclear, such as dachshund transcription factor 1 (DACH1), which normally functions as tumor suppressive protein by inhibiting the oncoprotein SNAI1 from transforming epithelial to mesenchymal cell, potentiating metastasis [15]. Thus anti-DACH1 is often found in paraneoplastic AE, also known as anti-neuronal nuclear antibodies type 3 (ANNA-3) [16]. ANNA-1 targets another intranuclear antigen, HuD, an RNA-binding protein that binds BDNF and Akt [17]. ANNA-2 is another RNA-binding protein known as NOVA. NOVA binds to pre-mRNA of glycine receptor [18,19]. Anti-Yo targets cerebellar degeneration-related protein 2 (CDR2), a leucine-zipper containing cytoplasmic protein that binds c-Myc, involved in mitosis [20]. Anti-Yo is often paraneoplastic and presents as cerebellar degeneration. Other subsets of autoantibodies target more accessible cell surface targets, mainly receptors, and ion channels. These include voltage-gated potassium channel (VGKC) and proteins associated with it, such as contacting associated protein 2 (CASPR2) and leucine-rich glioma inactivated 1 (LGI1), which helps to connect presynaptic and postsynaptic neurons through ADAM proteins. Glutamate, a major excitatory neurotransmitter, has several ionotropic and metabotropic channels targeted to several autoantibodies. Most famously, the anti-N-methyl D-aspartic acid (NMDA) receptor and α-amino-3-hydroxy-5-methyl-4-isoxazole propionic acid (AMPA) receptor are shown in the figure. In addition, a recently discovered anti-DPPX targets the CD26-like protein associated with Kv4 channels [21]. Other subsets of targets not shown here are proteins expressed on glial cells, such as aquaporin 4 in neuromyelitis optica spectrum disorders (NMOSD), anti-MOG in myelin oligodendrocyte glycoprotein antibody-associated disease (MOGAD), and anti-glial fibrillary acidic protein (GFAP). In adults, anti-GAD, anti-NMDAR, and anti-LGI1 constitute up to 65% of all AE. In pediatric cases, anti-NMDAR, anti-GAD, and anti-MOG constitute up to 92% of all AE.

Anti-GAD-65 Ab, which was weakly positive in our patient who developed recent T1DM diabetes, is associated with endocrine diseases, including type 1 diabetes mellitus, thyroiditis, vitiligo, celiac disease, and pernicious anemia, and neurologic syndromes including stiff-person syndrome, AE, autoimmune ataxia, brain stem encephalitis, autoimmune epilepsy, autoimmune myelopathy [22,23]. High GAD titers generally greater than or equal to 20.0 nmol/L were associated with specific neurological diseases compared with moderate-low titers in atypical syndromes and very low titers in DM-1 [22]. GAD involves synthesizing GABA by decarboxylation of glutamate, which requires a co-factor, pyridoxal 5’-phosphate. "GABA-ergic" nerve cells are where GAD and GABA are mainly present but are also detected in certain non-neuronal organs such as the pancreas, where GABA is stored in synaptic-like vesicles in islet beta cells. Although its functional role is unclear, it may be associated with paracrine effects of modulating glucagon secretion in alpha cells [24]. Despite studies in both in vitro and in vivo environments, the pathophysiological role of anti-GAD antibodies is unclear, suggesting the possibility of other autoantibodies targeting inhibitory synaptic antigens leading to hyperexcitability in GAD antibody-spectrum disorders [23]. Patients with the clinical picture of AE but are negative of characterized autoantibodies, as our patient, are defined as seronegative AE [2]. However, as new characteristic autoantibodies are discovered, the term "seronegative" may once become obsolete.

Another autoimmune condition highly prevalent in patients with autoimmune diabetes is hypothyroidism (Hashimoto thyroiditis), which can be associated with a neurological syndrome called Hashimoto encephalitis on rare occasions. Our patient had normal thyroid function, with negative antibodies for thyroid peroxidase, ruling out Hashimoto encephalopathy. Furthermore, Hashimoto encephalopathy is often steroid responsive [25].

Magnetic resonance imaging (MRI) is often used as a diagnostic imaging tool for AE. Involvement of bilateral medial temporal lobes on T2-weighted fluid-attenuated inversion recovery (FLAIR) MRI comprises one of the criteria for definite diagnosis of limbic encephalitis even in settings of negative autoantibody [2]. Other MRI patterns, including cortical/subcortical, striatal, diencephalic, and brainstem, can support possible or probable AE [1,2]. MRI of the head is abnormal in 30% of affected patients, mainly showing increased FLAIR signal involving the cortical, subcortical, or cerebellar regions [26]. Normal brain MRI cannot rule out AEs, and single-photon emission computed tomography (SPECT) or positron emission tomography (PET) scans are more sensitive in detecting dysfunction [27]. CT chest, abdomen, and pelvis, mammogram and breast MRI, testicular ultrasound, pelvic MRI, and whole body fluorine-18 fluorodeoxyglucose-positron emission tomography (^18^F FDG-PET) are often used to rule out underlying malignancies that may cause paraneoplastic etiologies of AE [28].

Initial treatment of AE includes intravenous immunoglobulin (IVIG), plasmapheresis, and steroids [29]. In a survey of the Autoimmune Encephalitis Alliance Clinicians Network (AEACN), 84% of responders preferred corticosteroids as initial monotherapy [28]. Since the pathophysiology of AE involves antibody production and inflammatory changes which occur behind the blood-brain barrier, there is often limited effectiveness of plasma exchange and IVIG, in contrast to a better response in systemic antibody-mediated diseases such as myasthenia gravis to the same modalities (PLEX and IVIG) [1]. Therapeutic plasma exchanges in refractory autoimmune encephalitis had been successful in around 50% of patients (predominantly anti-NMDAR encephalitis) [30]. Response of seronegative autoimmune encephalitis to IVIg has been recently reported to be favorable in most cases and one-third of patients as monotherapy, yet few may require other lines of treatment [31]. Second-line agents include alkylating agents, such as cyclophosphamide, often avoided due to toxicity, and B-cell depleting agents, such as anti-CD20 rituximab. Second-line agents are used in cases where clinical or radiological responses to first-line therapy are unsatisfactory after two to four weeks of treatment [26]. Rituximab has been increasingly used as an initial treatment as it is effective in refractory AE, reducing the risk of a clinical relapse [32]. In a German cohort, the success rate of rituximab in various autoimmune encephalitis ranged between 43% and 57% [33,34]. However, some patients might respond poorly to immunotherapy or require intensive and prolonged therapies [26].

"It would be dysteleological in the highest degree if self-poisons of the parenchyma-autotoxins-were formed," argued German physician, scientist, and Nobel Laureate Paul Ehrlich (1854-1915) regarding the possibility of autoimmunity in the explanation of certain encephalitides and hemolytic anemias. The following century witnessed an accumulation of evidence for autoimmunity and the "horror autotoxicus" as the etiologic basis of several diseases, including dozens of syndromic autoimmune encephalitides, some of which are of known antigenic targets, and many are yet to be discovered [35].

## Conclusions

In conclusion, we present a case that emphasizes the importance of considering seronegative autoimmune encephalitis in the context of a patient with other autoimmune diseases and no better explanation for their neurological manifestations. We highlight the importance of empiric treatment in such cases with immune suppressive treatment after excluding infectious etiologies. Such treatment can be life-saving and life-changing for those afflicted with the horror autotoxicus of a self-reactive immune system.
